# High dose of vesicular stomatitis virus-vectored Ebola virus vaccine causes vesicular disease in swine without horizontal transmission

**DOI:** 10.1080/22221751.2021.1903343

**Published:** 2021-04-02

**Authors:** Igor Morozov, Thomas P. Monath, David A. Meekins, Jessie D. Trujillo, Sun-Young Sunwoo, Kinga Urbaniak, In Joong Kim, Sanjeev K. Narayanan, Sabarish V. Indran, Wenjun Ma, William C. Wilson, Cassandra O'Connor, Sheri Dubey, Sean P. Troth, Beth-Ann Coller, Richard Nichols, Brian K. Martin, Heinz Feldmann, Juergen A. Richt

**Affiliations:** a Department of Diagnostic Medicine/Pathobiology, Center of Excellence for Emerging and Zoonotic Animal Diseases (CEEZAD), College of Veterinary Medicine, Kansas State University, Manhattan, KS, USA; b Bioprotection Systems, Inc, a subsidiary of NewLink Genetics Corp, Ames, IA, USA; c Center for Grain and Animal Health Research, Arthropod-Borne Animal Diseases Research Unit, Agricultural Research Service, United States Department of Agriculture, Manhattan, KS, USA; d Battelle Memorial Institute, Columbus, OH, USA; e Merck & Co, Inc., Kenilworth, NJ, USA; f Laboratory of Virology, Division of Intramural Research, National Institute of Allergy and Infectious Diseases, National Institutes of Health, Hamilton, MT, USA

**Keywords:** Ebola virus disease, VSV, virus-vectored vaccine, safety study, swine

## Abstract

The recent impact of Ebola virus disease (EVD) on public health in Africa clearly demonstrates the need for a safe and efficacious vaccine to control outbreaks and mitigate its threat to global health. ERVEBO® is an effective recombinant Vesicular Stomatitis Virus (VSV)-vectored Ebola virus vaccine (VSV-EBOV) that was approved by the FDA and EMA in late 2019 for use in prevention of EVD. Since the parental virus VSV, which was used to construct VSV-EBOV, is pathogenic for livestock and the vaccine virus may be shed at low levels by vaccinated humans, widespread deployment of the vaccine requires investigation into its infectivity and transmissibility in VSV-susceptible livestock species. We therefore performed a comprehensive clinical analysis of the VSV-EBOV vaccine virus in swine to determine its infectivity and potential for transmission. A high dose of VSV-EBOV resulted in VSV-like clinical signs in swine, with a proportion of pigs developing ulcerative vesicular lesions at the nasal injection site and feet. Uninoculated contact control pigs co-mingled with VSV-EBOV-inoculated pigs did not become infected or display any clinical signs of disease, indicating the vaccine is not readily transmissible to naïve pigs during prolonged close contact. In contrast, virulent wild-type VSV Indiana had a shorter incubation period and was transmitted to contact control pigs. These results indicate that the VSV-EBOV vaccine causes vesicular illness in swine when administered at a high dose. Moreover, the study demonstrates the VSV-EBOV vaccine is not readily transmitted to uninfected pigs, encouraging its safe use as an effective human vaccine.

## Introduction

Ebola virus disease (EVD) is an acute illness that can result in severe and highly lethal hemorrhagic fever [1]. EVD is caused by the highly pathogenic Ebola virus (EBOV), a member of the *Filoviridae* family [2]. The 2013–2016 west-African Ebola virus epidemic was the largest outbreak ever recorded, with over 28,000 cases and 11,000 deaths, and exposed an urgent need for vaccination strategies to control future outbreaks [3,4]. The outbreak prompted the progression of a promising Vesicular Stomatitis Virus (VSV)-vectored Ebola virus vaccine (designated V920, VSV-EBOV) through clinical trials [5–11]. Moreover, the VSV-EBOV vaccine, approved by the FDA and EMA in late 2019 and known as ERVEBO®, is being deployed under the Expanded Access clinical protocol to manage the ongoing EBOV outbreak in the Democratic Republic of the Congo (DRC) [12–14]. The vaccine is currently licensed in the US, EU, DRC, Burundi, Ghana, and Zambia.

VSV-EBOV consists of an attenuated recombinant Vesicular Stomatitis virus (rVSV) Indiana subtype backbone in which the VSV surface glycoprotein (VSV-G) has been replaced with the *Zaire ebolavirus* (representative EBOV) glycoprotein (GP) [15]. The vaccine provides complete protection against lethal EBOV challenge in rodents and non-human primates and has partial post-exposure efficacy [7,9,16]. VSV-EBOV has been administered to over 15,000 people in Phase 1, 2, and 3 clinical trials during the West African epidemic and has been administered to more than 250,000 people in the ongoing DRC outbreaks as of May 20, 2020 [8,12,14,17]. These trials, combined with pre-clinical studies, indicate that CD4+ T-cell-mediated antibody responses play a crucial role in protection of VSV-EBOV-vaccinated individuals against EVD and that the vaccine is generally well tolerated with limited adverse effects [12,14,18–21]. VSV-EBOV is therefore a highly promising tool currently being used to combat EVD and provides confidence in the future implementation of additional VSV-vectored vaccines in development [22–25].

VSV is a single stranded negative sense RNA virus and a member of the *Rhabdoviridae* family [26,27]. VSV causes Vesicular Stomatitis (VS), a disease that affects equids, cattle, and swine and is characterized by vesicular lesions, papules, erosions, and ulcers localized on the oral mucosa, snout, coronary bands of the feet, and/or teats [26–28]. The disease in livestock is not lethal, and the lesions usually resolve in under two weeks without complication [26–28]. Clinical signs of VSV infections are indistinguishable from other vesicular diseases, notably foot-and-mouth disease (FMD), which complicates epidemiological surveillance [29]. VS is endemic from northern South America through southern Mexico and causes sporadic epidemics in central/northern Mexico and the United States, with an outbreak in the United States ongoing as of September 2020 [28–31]. Transmission of VSV occurs through insect vectors, both biologically and mechanically, or through direct contact with open lesions [29,32–34]. Humans can be infected with VSV, but infection is usually asymptomatic or causes a limited, mild, influenza-like illness [35–38].

 VSV-Indiana has been established as a robust vaccine vector backbone for infectious diseases for well over a decade. A potential complication of using VSV-vectored vaccines is the unintentional introduction of the attenuated virus into livestock herds susceptible to VSV. This type of spillover event could complicate efforts to monitor and control vesicular diseases, particularly FMD, and could severely hinder the widespread implementation of VSV-vectored vaccines. VSV-G has been shown to be the main determinant for pathogenesis [39], however the effect of replacing it with the *Zaire ebolavirus* (EBOV) GP in the VSV-EBOV vaccine in livestock species has not been established. Pigs are susceptible to experimental infection with EBOV [40,41]. Moreover, a close relative of EBOV within the *Ebolavirus* genus, Reston virus, causes natural infection of pigs in the Philippines [42], and antibodies to ebolaviruses have also been found in pigs in Africa [43]. Therefore, clinical and pathological studies must be performed to determine the effects of infection in VSV-susceptible livestock by the VSV-EBOV vaccine virus and its potential for transmission within a herd [44].

Here, we demonstrate that administration of a high dose of VSV-EBOV does indeed cause overt VSV-like vesicular disease in swine, with vesicular lesions observed at the injection site and distal sites in a proportion of inoculated pigs. We also demonstrate that the VSV-EBOV vaccine is not readily transmissible to naïve pigs via direct contact over a prolonged period of time. In contrast, virulent wild-type VSV-Indiana (wtVSV) had a shorter incubation period in principal infected pigs and is easily transmitted to co-housed contact pigs. These results establish the clinical profile of VSV-EBOV/ERVEBO® vaccine virus infection in swine and provide evidence of its safety with regard to its transmission potential within agricultural species.

## Materials and methods

### Animals and housing

All work involving wtVSV and VSV-EBOV was performed under biosafety level 3Ag conditions at the Biosecurity Research Institute at Kansas State University (KSU). Animal research was conducted in compliance with the Animal Welfare Act and other federal statutes and regulations relating to animals and animal experiments under the protocol no. 3655, approved by the Institutional Animal Care and Use Committee (IACUC) at KSU on 2/02/2016.

Twenty-four (24) American Yorkshire/Landrace Crossbred weaned pigs, 4–5 weeks of age at the start of the study and of both sexes, were used for the study. Pigs were randomly assigned to one of four groups designated as Group 1 (VSV-EBOV-inoculated, *n* = 7), Group 2 (wtVSV-inoculated, *n* = 7), Group 3 (contact controls, *n* = 6) and Group 4 (non-inoculated negative controls, *n* = 4). Pigs were held five days prior to inoculation for acclimation and base-line observations. Group 1, 2, and 4 pigs were housed separately for the duration of the study. On 1 day post infection (DPI), contact control pigs of Group 3 were moved from the Group 4 room and evenly distributed (*n* = 3) in pens with Group 1 and Group 2 pigs.

### Viruses and virus titration

The clinical V920 vaccine virus (Lot 03 12 14) (VSV-EBOV; ERVEBO®) was provided by Bioprotection Systems and was manufactured using current good manufacturing practices (cGMP) by IDT Biologika (Dessau, Germany). wtVSV-Indiana virus, Strain L134-85 (wtVSV) was obtained from the Low Passage virus collection at the University of Texas Medical Branch. L134-85 was originally obtained from the mouth of a sick bovine in El Salvador in 1985. The virus was passaged twice in Vero cells, aliquoted, and refrozen at −80°C. Virus titration for wtVSV challenge stock was performed by TCID_50_ on Vero cells and virus titer was calculated using the method of Spearman and Karber [45].

### Inoculation of pigs and addition of contact controls

On 0 DPI, pigs were inoculated by both intradermal and intranasal routes. At the time of inoculation, the virus stock (VSV-EBOV or wtVSV) was allowed to slowly thaw at room temperature and was then maintained in an ice bath for no longer than 4 hours. Virus stocks were diluted in cold Minimum Essential Media (MEM) to achieve the desired dilutions. Pigs were sedated for virus inoculations by intramuscular injections of 2.0 mg/kg Telazol (Zoetis) and 0.5 mg/kg xylazine (Bayer).

For intradermal inoculation, pigs were injected in the apex of the snout (nasal planum) between the nostrils, taking care to ensure all inoculum was received. Following injections, the skin surface was swabbed with alcohol to decontaminate the area. Each pig received 2.0 × 10^7^ PFUs of respective virus material, administered in one (wtVSV) or two (VSV-EBOV) 0.1 mL injections calculated based on the titer of the stock virus. For intranasal inoculation the syringe tip, without a needle, was inserted into the pig’s nostril and the nostril squeezed around the syringe to form a seal. While the pig’s head was tilted up, the inoculum was then injected rapidly to coat the pig’s nasal turbinate and oropharynx. Each pig received 2.0 × 10^7^ PFUs in a 0.5 cc volume distributed equally between nostrils. Uninoculated and contact control pigs were not inoculated (mock infected) with MEM to avoid epidermal injury that could complicate clinical evaluations.

On 1 DPI, three non-inoculated contact pigs from Group 3 were randomly selected and added to each of the inoculated groups (Groups 1 and 2) to serve as contact controls. Baseline samples (blood, nasal and tonsillar swabs) from the contact pigs were collected prior to relocating.

### Clinical observations and recording

Post-inoculation, animals were observed for clinical signs of VSV infection, including vesicular lesions on the snout, lips, oral cavity and coronary bands, reluctance to eat due to vesicle-related discomfort, rectal temperature, activity, appetite, external lesions, and lameness. Statistical significance of increased rectal temperatures was determined using a student’s *t*-test.

Lesion locations were recorded and photographed and an overall lesion score per pig was assessed daily according to the following VSV-specific scale modified from [39]: 0 = No visible lesion; 1 = <2 cm diameter lesion at inoculation site; 2 = >2 cm diameter lesion at inoculation site and/or multiple lesions at inoculation site; 3 = <2 cm diameter lesion at non-inoculation site: 4 = >2 cm diameter lesion at non-inoculation site and/or multiple lesions.

### Ante-mortem sample collection

Blood and nasal/tonsillar swabs were collected from pigs in all three groups. Sample collection was facilitated by sedating the pigs on collection days. Blood samples were collected from each pig and processed for virus neutralization (VN) titers and/or PRNT_60_. Nasal and oral/tonsillar swabs were collected from each animal for RT-qPCR analysis. Tonsil fluid was collected by gently scraping the tonsil with a long-handled spoon followed by fluid collection with a swab. External lesions were swabbed for RT-qPCR when they were first noted to be open or weeping. RNA from clinical samples was preserved by collecting samples in RNA*later* (Qiagen) in an RNase-free tube, which was kept at 4°C overnight then transferred to −80°C until shipped.

### Terminal sacrifices, necropsy, and tissue analysis

On 2 and 10 DPI, two randomly selected animals from each inoculated group (Groups 1 and 2) and one negative control pig (Group 4) were euthanized and necropsied, with the remaining three inoculated pigs in each group euthanized and necropsied on 20 DPI (Group 4) or 21 DPI (Groups 1, 2, and contact controls).

Humane euthanasia was completed through intravenous injection of Sleepaway (Zoetis) according to the IACUC protocol. During necropsy, the following tissue samples were collected: grossly visible external lesion tissue; intradermal injection site (nasal planum); lymph nodes (mandibular, parotid, retropharyngeal, superficial cervical); tonsil; testicle; Peyer’s patch/ileum; kidney; parotid salivary gland; mammary gland; gluteal skeletal muscle; lung; spleen; urinary bladder; and any other tissue with a grossly-visible lesion. The lymph nodes tested were chosen based on their anatomic location with respect to lymphatic drainage of the inoculation site. Tissue samples were placed in RNA*later* (Qiagen) for RT-qPCR analysis. For immunohistochemistry (IHC) examination, select samples were placed in 10% neutral buffered formalin for 20–30 days.

### Quantitation of wtVSV or VSV-EBOV virus RNA in samples by RT-qPCR

The following samples were tested for the presence of wtVSV or VSV-EBOV nucleoprotein RNA via RT-qPCR: any external or oral lesion swabs collected ante-mortem; nasal and tonsillar swabs; sera samples; and tissues collected at necropsy. The RT-qPCR testing for all samples was conducted at Battelle Memorial Institute (West Jefferson, OH) using a qualified RT-qPCR assay for the VSV nucleocapsid protein (NP) gene with the group association of samples blinded. Viral RNA from all study samples was isolated using the QIAamp *cador* Pathogen Mini kit (Qiagen). A positive RNA extraction control containing VSV-EBOV and a negative extraction control were included in each batch of samples to confirm successful isolation of genetic material and to rule-out cross contamination. RT-qPCR was run on the StepOnePlus Real-Time PCR System (Applied Biosystems, USA) using the Ultrasense One-Step Quantitative RT–PCR system (Invitrogen, USA) for master mix preparation and a custom primer-probe mix targeting a section of the VSV NP gene (Integrated DNA Technologies, USA). Sequences for the primers and probe were obtained from Gunther, et al [46] and optimized at Battelle for use in the VSV NP RT-qPCR reaction.

Forward Primer: 5’-GACCTTGTATCCTTGAAAGCC-3’

Reverse Primer: 5’-CATTTGTGTTCTGCCCACTC-3’

Probe: 5’-6-FAM-TGCTTCCAG/ZEN/AACCAGC GCAGATGACAAA-3’-1ABkFQ

The primer-probe mix containing these oligos was prepared for final concentrations of 300nM forward primer, 600nM reverse primer, and 250nM probe in the RT-qPCR reaction. A synthetic RNA fragment (Biosynthesis, USA) containing the VSV NP amplicon (150 bp) diluted over a range from 2 × 10^6^ copies/µL to 0.2 copies/µL was used as the reference standard. Each RT-qPCR plate included a qualified reference standard dilution series in triplicate with each test sample, isolation negative and positive controls, and no template controls loaded in duplicate. All samples and controls were loaded to the RT-qPCR in 5 µL for a total reaction volume of 25 µL per well. Porcine biological matrices did not have an inhibitory effect on the RT-qPCR except for the tonsil tissue. Tonsil RNA was therefore diluted 1:10 immediately prior to loading on the plate in order to allow accurate quantitation of the VSV NP target.

### Serum neutralization titers

VSV and VSV-EBOV VN titers were determined in sera collected on 0, 10, and 21 DPI. The VSV-EBOV plaque reduction neutralization test (PRNT_60_) was conducted at Q^2^ Solutions-Vaccines (Morrisville, NC) as described previously [20]. Determination of the 60% neutralizing titer (PRNT_60_) is based upon the percent reduction in viral plaques in the presence of serum compared to that of the virus control without serum. The VSV serum neutralization assay was conducted at KSU. VN was performed using approximately 90% confluent Vero E6 cells via serial dilution of serum, pre-incubation of serum with homologous virus, inoculation onto cells, incubation and detection. A 10^3^ TCID_50_ VSV sample was added to serial two-fold dilutions of serum and incubated for 1 hour prior to inoculation onto Vero E6 cells. Detection of virus neutralization was determined approximately 48 hours post-inoculation by the absence of cytopathic effect (CPE) in the virus-inoculated wells. End-point neutralizing antibody titers of test sera were determined as the highest dilution of serum that prevents virus infection (CPE) in inoculated wells.

### Histopathology and immunohistochemistry

Formalin-fixed tissues were paraffin-embedded, sectioned, and stained with hematoxylin and eosin (HE). Following histopathology, IHC was performed with anti-VSV-G rabbit polyclonal (Alpha Diagnostic, San Antonio, TX) or anti-Ebola-GP rabbit polyclonal antibodies (IBT Bioservices, Gaithersburg, MD). Antigen retrieval was performed for 20 minutes at 100°C with pH 9.0 EDTA. The primary antibody and AP anti-rabbit IgG polymer were incubated for 15 and 30 minutes at ambient temperature, respectively. The slides were then developed using the Bond Polymer Refine Red Detection Kit on an automated Leica Bond IHC Stainer (Leica Biosystems, Buffalo Grove, IL) and were counterstained with hematoxylin.

## Results

### Outline of study design

To determine the effects of the VSV-EBOV vaccine virus after infection of pigs, two groups of swine were housed separately and inoculated by intranasal and intradermal (nasal planum) routes with a total of 4.0 × 10^7^ plaque-forming units (pfu) of either wild-type VSV Indiana L134-85 (wtVSV, *n* = 7) or a clinical lot of the ERVEBO® vaccine (VSV-EBOV, *n* = 7) (Table S1). To monitor virus transmission, three uninoculated contact control pigs were introduced to the wtVSV and VSV-EBOV treatment groups, respectively, one day post inoculation (DPI). Four additional environmental control pigs were housed separately as uninoculated negative controls for the duration of the study. Clinical signs were observed, and samples were collected throughout the study as outlined in the Materials and Methods.

### High dose VSV-EBOV vaccine causes vesicular disease in swine

**Figure 1.**
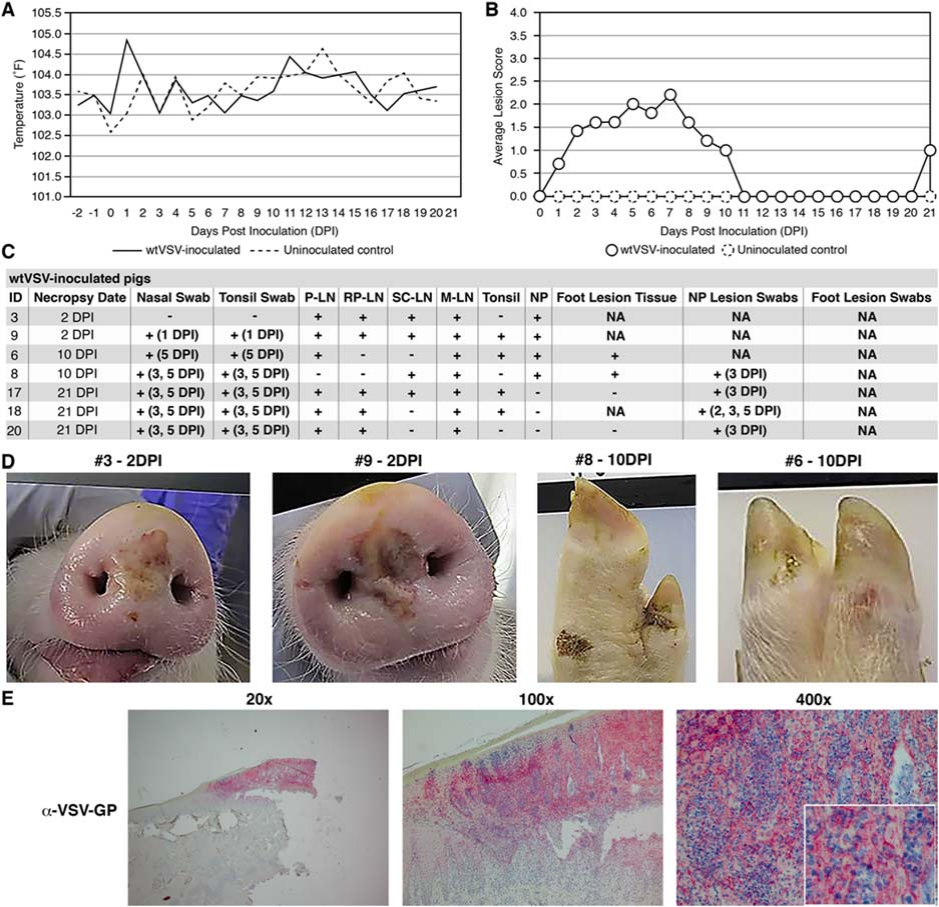
Clinical analysis of wtVSV-inoculated pigs. (A) Average daily temperatures of wtVSV-inoculated pigs compared to uninoculated pigs. (B) Average lesion scores of wtVSV-inoculated pigs compared to uninoculated environmental controls on each day of the study. (C) Summary of RT-qPCR analysis performed to detect viral RNA in clinical samples collected throughout the study, indicating the presence (+) or absence (−) of viral RNA. P-LN – parotid lymph node; RP-LN – retropharyngeal lymph node; SC-LN – superficial cervical lymph node; M-LN – mandibular lymph node; NP – nasal planum; NA – Not Applicable because not present or collected; DPI - days post inoculation in which positive samples were detected. (D) Representative pictures showing vesicular lesions in pigs. (E) Immunohistochemistry analysis performed on nasal planum lesion tissue collected on 2 DPI necropsy from wtVSV-infected pig #3 showing positive (red) immunostaining using anti-VSV-G rabbit polyclonal antibody localized to the stratum spinosum and granulosum of the epidermis.

As expected, wtVSV inoculated pigs in Group 2 displayed signs consistent with vesicular disease (Figure 1). This group exhibited an elevated average body temperature compared to uninoculated controls (*p* = <0.01) at 1 DPI before returning to normal for the remainder of the study (Figure 1(A)). Vesicular lesions were first observed at the inoculation site (nasal planum) on 1 DPI and all inoculated pigs developed vesicular lesions at the inoculation site by 2 DPI (Figure 1(B), Table S2). The peak of lesions in this group was observed between 3 and 8 DPI (Figure 1(B), Table S2). Viral RNA was detected in lesion swabs collected from four different pigs between 2 and 5 DPI, confirming that the lesions are due to VSV infection (Figure 1(C), Table S3). Nasal and tonsillar swabs also indicated the presence of viral RNA for the remaining pigs at 5 DPI, although no viral RNA was detected in sera (Figure 1(C), Tables S4 and S5). During scheduled necropsy on 2 DPI, both pigs (#3 and #9) had significant vesicular lesions (>2 cm) at the nasal planum (inoculation site) that tested positive for viral RNA (Figure 1(C,D), Table S6). The presence of VSV-G antigen was confirmed in tissue from the nasal planum lesion of pig #3 using IHC (Figure 1(E), Figure S1). One pig (#8) developed significant lesions at a distal site (foot), which were observed between 5 and 10 DPI (Figure 1(D), Table S2). A small lesion was also observed on the foot of another pig (#6) on 10 DPI (Figure 1(D), Table S2). Tissue collected from these foot lesions during 10 DPI necropsy tested positive for viral RNA (Figure 1(C), Table S7). All lesions in the wtVSV inoculated group were fully resolved by 11 DPI and no active lesions were observed on 21 DPI necropsy (Figure 1(B), Table S2). Viral RNA was widely detected in lymphoid and nasal planum tissues collected on each necropsy day (Figure 1(C), Table S6). As expected, all remaining wtVSV inoculated pigs seroconverted by 21 DPI with a geometric mean of VN titers measuring 6,942 (Table S8). Uninoculated negative control pigs did not exhibit any vesicular lesions at any point throughout the study (Figure S2).

**Figure 2.**
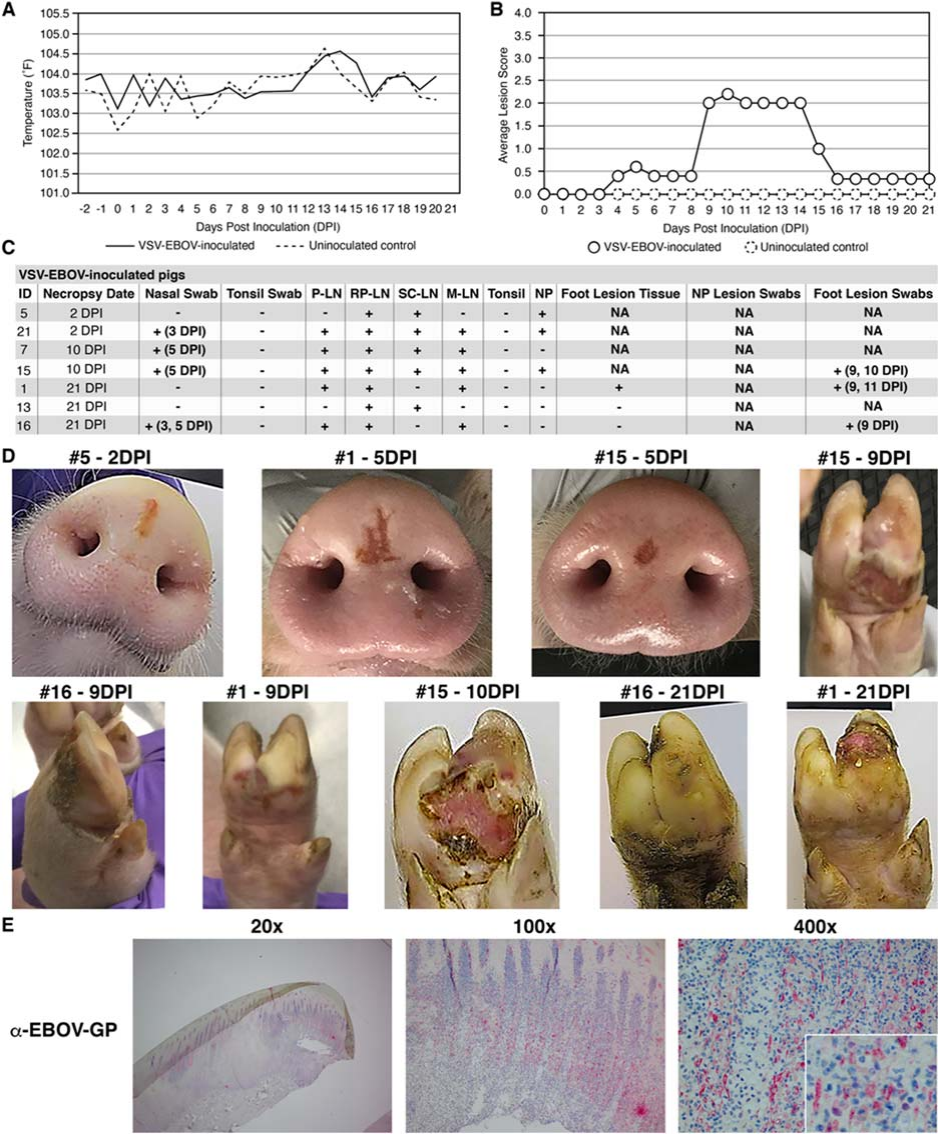
Clinical analysis of VSV-EBOV-inoculated pigs. (A) Average daily temperatures of VSV-EBOV-inoculated pigs compared to uninoculated controls. (B) Average lesion scores of VSV-EBOV-inoculated pigs compared to uninoculated controls on each day of the study. (C) Summary of RT-qPCR analysis performed to detect viral RNA in clinical samples collected throughout the study, indicating the presence (+) or absence (−) of viral RNA. P-LN – parotid lymph node; RP-LN – retropharyngeal lymph node; SC-LN – superficial cervical lymph node; M-LN – mandibular lymph node; NP – nasal planum; NA – Not Applicable because not present or collected; DPI - days post inoculation in which positive samples were detected. (D) Representative pictures showing vesicular lesions in VSV-EBOV-inoculated pigs. (E) Immunohistochemistry analysis performed on foot lesion tissue (hoof and skin) collected on 10 DPI necropsy from VSV-EBOV-inoculated pig #15 showing positive (red) immunostaining using anti-EBOV-GP rabbit polyclonal antibody localized to the stratum spinosum and granulosum of the epidermis.

The VSV-EBOV/ERVEBO® vaccine also caused vesicular disease in pigs, but with some notable differences in clinical signs compared to wtVSV (Figure 2). In contrast to wtVSV-inoculated pigs, VSV-EBOV-inoculated pigs did not exhibit elevated average body temperatures at any point during the study, except for an isolated increase on 14 DPI (*p* ≤ 0.01) (Figure 2(A)). VSV-EBOV-inoculated pigs developed VSV-like lesions of similar severity and anatomical distribution as observed with wtVSV-inoculated pigs, although the lesions appeared later than with wtVSV inoculated pigs with peak average lesion scores between 9 and 14 DPI (Figure 2(B), Table S2). On 2 DPI necropsy, a small red-tan linear lesion was observed at the nasal planum inoculation site of pig #5 (Figure 2(D)). RT-qPCR and IHC analysis confirmed that the lesion on pig #5 contained viral RNA and the EBOV-GP antigen (Figure 2(C), Figure S3, Table S6). Viral RNA was also detected in other tissues from pig #5 namely in two lymph nodes as well as the gluteal muscle, Peyer’s patch, and the testicle (Figure 2(C), Table S6). Two of the VSV-EBOV-inoculated pigs (#1 and #15) developed lesions at the injection site by 4 and 5 DPI (Figure 2(D)). By 9 DPI, pigs #1, #15, and #16 developed distal lesions on their feet that tested positive for viral RNA (Figure 2(C,D), Table S3). Pig #15 was sacrificed for scheduled necropsy on 10 DPI and exhibited small skin erosions on the nasal planum, intact vesicular lesions on the right front and both rear feet, and a severe ulcerated lesion on the left front foot (Figure 2(D)). IHC confirmed the presence of EBOV-GP antigen in the ulcerated foot lesion tissue from pig #15 (Figure 2(E), Figure S4). Viral RNA was also detected in pig #15 in all four tested lymph nodes, as well as the testicle, urinary bladder, and parotid salivary gland (Figure 2(C), Table S6). The lesions in Pig #1 and Pig #16 persisted throughout 21 DPI, with the highest gross lesions scores documented between 9 and 15 DPI (Figure 2(B), Table S2). On 21 DPI necropsy, a severe ulcerated lesion with sloughing of the foot nail was observed in Pig #1, and a healed erosion lesion on the left rear foot of Pig #16 (Figure 2(D)). Viral RNA was detected in the spleen of Pig #16 and in three lymph nodes in both Pig #1 and Pig #16 (Figure 2(C), Table S6). Importantly, three out of seven VSV-EBOV-inoculated pigs (#7, #13, #21) did not display any obvious signs of vesicular disease throughout the study, although viral RNA was detected in the lymph nodes of each of these pigs (Figure 2(C), Table S6). Viral RNA was detected in nasal swabs on 3 DPI (Pigs #16 and #21) and 5 DPI (pigs #7, #15, #16) (Figure 2(C), Table S4). Interestingly, no viral RNA was detected in any tonsillar swabs or tonsil samples from any VSV-EBOV-inoculated pigs throughout the study; this is in contrast to wtVSV-inoculated pigs (Figure 2(C), Table S4). No viral RNA was detected in sera of VSV-EBOV-inoculated pigs (Table S5). All VSV-EBOV-inoculated pigs seroconverted by 10 or 21 DPI with average VSV-EBOV PRNT_60_ titers of 373 (Table S9), suggesting protection of vaccinated pigs from EVD. These results indicate that a high dose of experimentally inoculated VSV-EBOV can cause vesicular disease in pigs which is similar to wtVSV, with notable differences in average body temperatures, time of onset of vesicle formation, a lack of viral replication in tonsillar tissue, and an absence of vesicular disease in several pigs (three out of seven).

### VSV-EBOV vaccine virus is not readily transmissible to contact pigs

**Figure 3.**
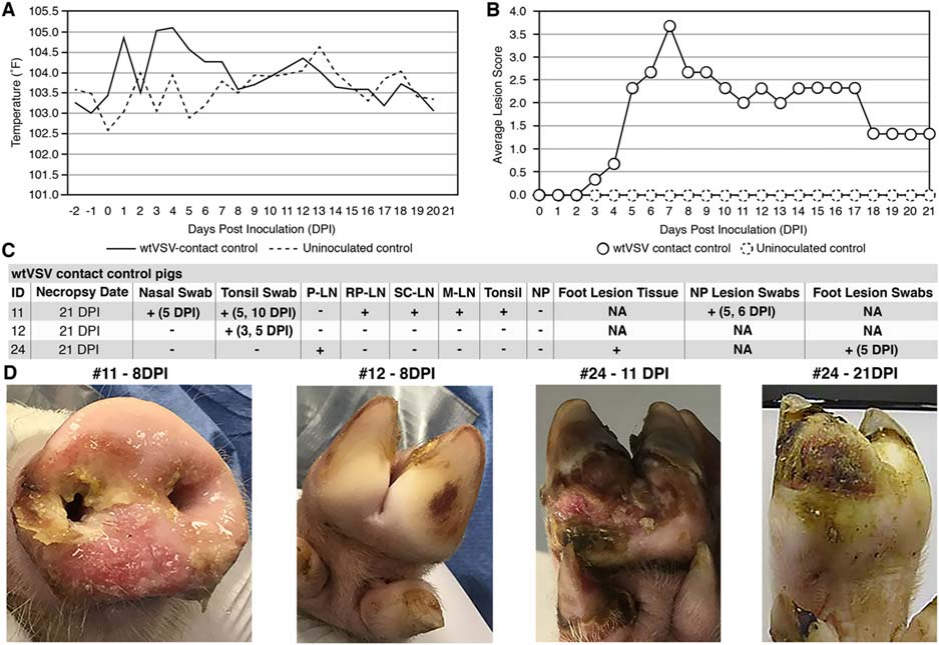
Clinical analysis of wtVSV-contact control pigs. (A) Average daily temperatures of wtVSV- contact control pigs compared to uninoculated controls. (B) Average lesion scores of wtVSV-contact control pigs compared to uninoculated controls on each day of the study. (C) Summary of RT-qPCR analysis performed to detect viral RNA in clinical samples collected throughout the study, indicating the presence (+) or absence (−) of viral RNA. P-LN – parotid lymph node; RP-LN – retropharyngeal lymph node; SC-LN – superficial cervical lymph node; M-LN – mandibular lymph node; NP – nasal planum; NA – Not Applicable because not present or collected; DPI - days post inoculation of principal infected pigs in which positive samples were detected in this group. (D) Representative pictures showing vesicular lesions in wtVSV-contact control pigs.

The wtVSV contact control pigs displayed clinical signs of vesicular disease similar to the principal infected group, demonstrating the transmission potential for wtVSV (Figure 3). The average body temperature of wtVSV contact animals was elevated compared to uninoculated controls between 4 and 7 DPI, i.e. between 3- and 6-days post contact (DPC) (*p* ≤ 0.01), before returning to normal (Figure 3(A)). Two of the three contact pigs (#11 and #24) developed lesions characteristic of VSV infection (Figure 3(B,D), Table S2). Pig #11 developed severe lesions on its snout which also spread into the oral mucosa between 3 and 13 DPI and resolved by 21 DPI (Figure 3(D), Table S2). Pig #24 developed severe lesions on its feet which were detected from 5 to 21 DPI (Figure 3(B,D), Table S2). Lesion swabs from pigs #11 and #24 tested positive for viral RNA (Figure 3(C), Table S3). The remaining contact pig (#12) was mostly normal throughout the study and was observed with some suspect lesions on its foot only once on 8 DPI (Figure 3(D)). During the 21 DPI necropsy, a large area of sloughed skin was observed on the right rear foot of Pig #24 (Figure 3(D)). Importantly, tissues derived from the foot lesion of Pig #24 tested positive for viral RNA (Figure 3(C), Table S7). Viral RNA was not detected in any of the contact animals in tissues collected on 21 DPI from the gluteal muscle, Peyer’s patch, spleen, testicle, urinary bladder, parotid salivary gland, or nasal planum (Figure 3(C), Table S6). Isolated samples from nasal swabs, tonsillar swabs, tonsil, lymph nodes, and spleen contained viral RNA for Pigs #11 and #24 (Figure 3(C), Tables S4 and S6). Moreover, viral RNA was detected in the mammary gland and tonsillar swabs of Pig #12 despite the absence of overt vesicular lesions (Figure 3(C), Tables S4 and S6). No viral RNA was detected in sera of the contact pigs (Table S5). All three wtVSV contact pigs seroconverted by 21 DPI with a geometric mean of VN titers measuring 13,653 (Table S8). These data indicate that wtVSV is readily transmissible to uninfected contact pen mates when they are co-housed with infected pigs, and results in a disease progression similar to those experimentally inoculated with wtVSV.

**Figure 4.**
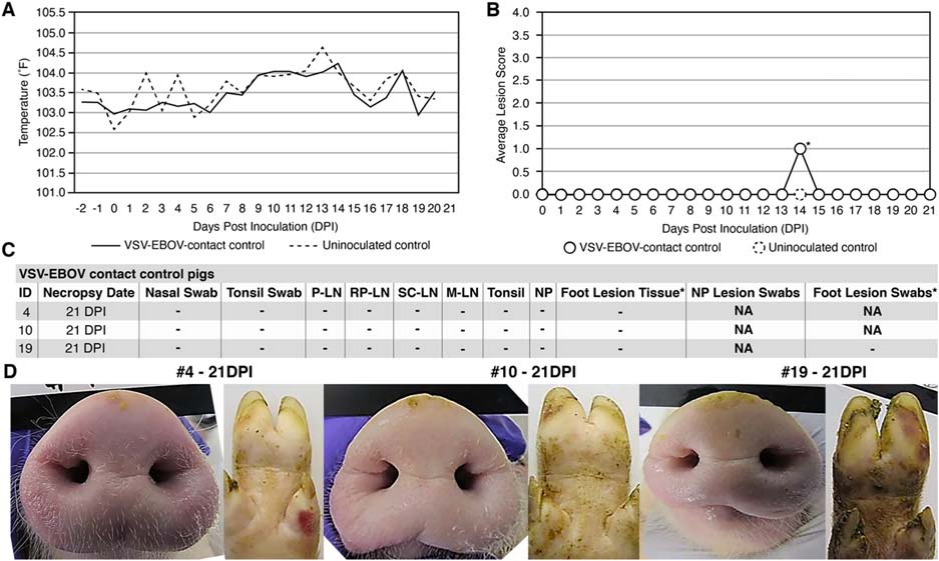
Clinical analysis of VSV-EBOV-contact control pigs. (A) Average daily temperatures of VSV-EBOV contact control pigs compared to uninoculated controls. (B) Average lesion scores of VSV-EBOV contact control pigs compared to uninoculated controls on each day of the study. * – one pig (#10) showed a focal <2 cm reddening on a hind limb on 14 DPI that was transient and did not show vesicular or ulcerative features typical of VSV infection and was ultimately determined to be a mechanical injury. (C) Summary of RT-qPCR analysis performed to detect viral RNA in clinical samples collected throughout the study, indicating the presence (+) or absence (−) of viral RNA. P-LN – parotid lymph node; RP-LN – retropharyngeal lymph node; SC-LN – superficial cervical lymph node; M-LN – mandibular lymph node; NP – nasal planum; NA – Not Applicable because lesions not present or collected; DPI - days post inoculation of principal infected pigs. * – suspected foot lesion swabs and suspected lesion tissues were collected on any tissue that was discolored even if the cause was likely a mechanical injury or benign discoloration and tested for viral RNA. All such samples were negative for VSV-specific RNA. (D) Representative pictures showing a lack of vesicular lesions in VSV-EBOV-contact control pigs.

In contrast, the three VSV-EBOV contact control pigs did not display any sign of vesicular disease or infection throughout the course of the study (Figure 4). Like the VSV-EBOV infected group, the average body temperature of VSV-EBOV contact pigs was not elevated at any point during the study (Figure 4(A)). Also, no significant lesions were observed for the VSV-EBOV contact animals throughout the study (Figure 4(B,D), Table S2). Pig #10 did show focal (<2 cm) reddening of a hind limb on 14 DPI, but it did not exhibit vesicular or ulcerative features typical of VSV. On 21 DPI necropsy, focal hemorrhages were noted on the feet of Pig #4, but there was no evidence of vesicles or ulcers characteristic of VSV infection and this was likely due to a mechanical trauma (Figure 4(D)). Viral RNA was not detected in any tissue, swabs, or sera collected from the VSV-EBOV contact pigs and they did not seroconvert to VSV-EBOV by the end of the study (Figure 3(C), Tables S5 and S9). These results reveal that the VSV-EBOV vaccine virus is not readily transmissible to naïve pigs which were kept in close prolonged contact, despite its ability to cause vesicular disease and virus shedding in principal inoculated pigs.

## Discussion

The VSV-EBOV vaccine has emerged as a highly promising EVD control strategy and is the most advanced vaccine currently available to mitigate the threat of EVD to human health [8]. Its ability to provide a high level of protection against EVD, combined with its limited side-effect profile, suggests it may become a mainstay in our response arsenal to combat future EVD epidemics. However, there are legitimate concerns regarding the potential for spillover to local livestock populations due to the VSV vector backbone. The infectivity of the VSV-EBOV vaccine virus in VSV-susceptible livestock and its potential for transmission therefore warrants close investigation.

VSV-EBOV vaccine virus infection causes obvious vesicular disease in swine under the experimental conditions of the present study. Of the seven pigs infected with a high dose of VSV-EBOV, four developed lesions on the nasal planum injection site and three of those (not sacrificed on 2 DPI) developed distal lesions on their feet. Some of these lesions were severe, including the coronary band lesion found on Pig #16, the vesicular lesion on the foot of Pig #1 that remained open at 21 DPI, and particularly the foot lesions found on Pig #15 that resulted in widespread sloughing of the skin. These results are unprecedented considering that no vesicular lesions were observed in multiple VSV-EBOV-infected rodent and non-human primate models [8,15,16,47,48]. Small mucosal lesions have been reported in humans in clinical trials but have not been documented virologically to be due to the vaccine virus. A small number of VSV-EBOV-vaccinated humans did developed transient vesicular or painless purpuric skin lesions, but these clinical signs were mild compared to those observed in pigs; they mainly occurred in human vaccinees with the highest dose tested, and are negligible considering the number of individuals who have received the ERVEBO® vaccine [20,21,49]. The present study therefore represents the first observation of overt vesicular disease elicited by the VSV-EBOV vaccine virus in any animal tested so far.

The vesicular disease observed in VSV-EBOV-inoculated pigs was not encountered in a previous pilot study, in which a lower dose of VSV-EBOV (1.0 × 10^6^ pfu, administered intradermally to piglets of the same age) failed to elicit vesicular lesions, viral replication in tissues, or seroconversion [50]. However, this previous study also failed to induce significant vesicular lesions or viral replication in tissues for pigs inoculated with wtVSV. The current study indicates that a higher inoculation dose (4.0 × 10^7^ pfu) applied simultaneously intradermally and intranasally is necessary to elicit vesicular lesions for both wtVSV and VSV-EBOV viruses. While transmission of VSV via direct contact between domestic livestock and humans has been demonstrated previously [36,37], the level of VSV-EBOV exposure required to produce vesicular disease in pigs is unlikely to occur from human contact in a natural setting. VSV-EBOV shedding from human vaccinees occurs in a low proportion of subjects and is likely to be at a low level based on the RT-qPCR Ct values obtained so far [20]. Moreover, the VSV-EBOV vaccine is unable to be transmitted via blood-feeding insect vectors [51]. Based on these studies, VSV-EBOV-induced vesicular disease in swine would require intradermal or intranasal injection or infection of abraded skin and would only cause overt illness in rather high doses. The low likelihood of such exposures of swine to the VSV-EBOV vaccine virus due to viral shedding from vaccinees, or any other means, during a closely monitored EVD outbreak and vaccination situation provides strong evidence for the safety of its use.

To date, it is unknown whether the clinical signs observed in swine infected with high doses of VSV-EBOV could be replicated in other VSV-susceptible livestock species such as horses and cattle. A study with a similar design to our study was conducted by Colorado State University in yearling mixed breed horses. The animals were infected with 10^7^ pfu of wtVSV (L134-85) or VSV-EBOV by the IN route (without scarification of the muzzle). None of the animals developed any lesions; however, the absence of disease in the wtVSV infected control horses did not allow a conclusion regarding susceptibility of horses to the vaccine virus (R.A. Bowen, Colorado State University, unpublished data, 2017).

A particularly intriguing feature of the clinical signs in VSV-EBOV-inoculated pigs is that the anatomic location of vesicular lesions (nasal planum injection site, feet) mirrored those found in wtVSV-inoculated pigs. This is interesting considering that VSV-G has been completely replaced by the EBOV-GP and both glycoproteins are important in the distinct tropism and pathogenicity of their respective parent viruses [39,52–55]. The observed pathology suggests that the anatomic expression of vesicular lesions in VSV-EBOV-inoculated pigs is primarily associated with the VSV-backbone. This is consistent with previous findings that VSV pseudotyped with EBOV-GP showed greater affinity for epithelial cells rather than the endothelial cells and hepatocytes preferentially infected by EBOV [56]. Moreover, the VSV-EBOV tropism observed in this study bore no similarity to the pathogenesis of EBOV infection in swine, which is generally limited to the respiratory system [40]. The retention of the VSV matrix (M) protein, known to be involved in VSV tropism [55], is most likely responsible for the observed pathological observations in VSV-EBOV-infected pigs. Determining the reason why the expression of EBOV-GP instead of VSV-G did not result in a novel phenotype in swine will require additional studies. It will also be important to determine, in future studies, whether the surface glycoproteins of other VSV-vectored vaccines (e.g. influenza virus HA, SARS-CoV-2 Spike, Lassa virus GPC) have a greater influence on tropism in susceptible species.

Although the lesions observed with VSV-EBOV mirrored that of wtVSV, there were several notable differences in the disease progression between the two viruses. Importantly, three out of seven pigs inoculated with VSV-EBOV did not develop any obvious vesicular clinical signs, despite a confirmation of infection via RT-qPCR and seroconversion titers. Conversely, all wtVSV-inoculated pigs developed vesicular lesions. wtVSV infection resulted in an increase in average body temperature immediately after infection, but no such increase was observed in VSV-EBOV-inoculated pigs. Moreover, the onset to vesicular disease was delayed by several days for VSV-EBOV-inoculated pigs. Lastly, no quantifiable VSV-EBOV RNA was found in any tonsil tissue samples or tonsillar swabs during the study. The reason for these differences may be due to a slower rate of replication for the VSV-EBOV vaccine virus when compared to the wtVSV. This possibility is consistent with the lower level of VSV-EBOV antigen expression detected in VSV-EBOV-induced lesions compared to VSV antigen expression detected in wtVSV-induced lesions.

The present study also provides strong and compelling evidence that the VSV-EBOV vaccine virus is not readily transmissible to naïve pen mates. No evidence of vesicular disease was observed in any of the co-housed contact pigs, despite being in close contact with VSV-EBOV-inoculated pigs for 20 days. Moreover, no quantifiable viral RNA was detected in any tissue or swab from the VSV-EBOV contact pigs and none of these pigs seroconverted. These results therefore provide compelling evidence that VSV-EBOV is not efficiently transmissible to naïve penmates. The mechanism underlying the lack of transmission of VSV-EBOV to other pigs remains unknown.

The transmission cycle of VSV is complex and poorly understood, although evidence shows that it is transmitted via a combination of biological vectors, mechanical vectors, and direct/indirect contact between animals, with contact transmission being dependent upon the presence of vesicular lesions [57–59]. Previous experimental studies involving VSV transmission indicate that it spreads quickly from infected animals to contact animals with a direct correlation between the appearance of vesicles on the principal infected pigs and the transmission event [32,34,60]. The current study effectively replicated the direct and indirect transmission of VSV, evidenced by the obvious signs of vesicular disease, widespread distribution of viral RNA, and seroconversion in the contact pigs. wtVSV contact control pigs actually had higher average lesion scores and higher VN titers than wtVSV-inoculated pigs, which suggests an efficient and prolonged period of transmission to the contact animals. Interestingly, open lesions were present for multiple days on both the nasal planum and feet of VSV-EBOV-inoculated pigs, providing ample opportunity for transmission to uninfected contact animals. The lack of spread of the VSV-EBOV vaccine, different from wtVSV, is consistent with results from non-human primates and humans, where there is limited viremia and low potential for shedding and transmission [16,20,21,49]. Additional studies will be necessary to understand the mechanism underlying the lack of transmission from VSV-EBOV-infected pigs with vesicular lesions. Regardless, the absence of transmission provides confidence that the VSV-EBOV vaccine virus is unlikely to spread if a spillover event occurs, and thus indicates a positive safety profile for the vaccine in general.

The present study provides comprehensive evidence indicating that VSV-EBOV inoculation at a high dose results in VSV-like vesicular disease in pigs but is unable to readily transmit to naïve pigs even after prolonged exposure. Overall, the study provides compelling evidence suggesting that the VSV-EBOV vaccine can be used safely to control EVD outbreaks in the future without concern for negative spillover effects in local livestock populations.

## Supplementary Material

Supplemental Material
