# Supplementary material for: High dose of vesicular stomatitis virus-vectored Ebola virus vaccine causes vesicular disease in swine without horizontal transmission
Source: Emerg Microbes Infect. 2021 Apr 2;10(1):651–63. doi: 10.1080/22221751.2021.1903343 (PMC8023602; doi:10.1080/22221751.2021.1903343)
Supplement: Supplemental Material [file TEMI_A_1903343_SM4064.pdf]

Supplemental Materials

Group	# Pigs	Treatment	Route (simultaneous)	Dose
1	7	recombinant V920 Ebola virus vaccine (VSV-EBOV)	Intradermal (ID)<br>Intranasal (IN)	ID = 2 x 10^7 pfu in 2 x 0.1cc<br>IN = 2 x 10^7 pfu in 0.5cc
2	7	wild type VSV<br>Indiana L134-85 (wtVSV)	Intradermal (ID)<br>Intranasal (IN)	ID = 2 x 10^7 pfu in 0.1cc<br>IN = 2 x 10^7 pfu in 0.5cc
3	6	none (contact controls)	NA	NA
4	4	none (negative controls)	NA	NA

Table S1. Study Design. Outline of the experimental design of the study. NA – not applicable.

	wtVSV-inoculated pigs				VSV-EBOV-inoculated pigs				wtVSV contact control pigs				VSV-EBOV contact control pigs		
DPI	No lesion (Score=0)	Lesions at inoc. Site (Score = 1-2)	Lesions at Distal Site (Score = 3-4)	DPI	No lesion (Score=0)	Lesions at inoc. Site (Score = 1-2)	Lesions at Distal Site (Score = 3-4)	DPI	No lesion (Score=0)	Lesions at inoc. Site (Score = 1-2)	Lesions at Distal Site (Score = 3-4)	DPI	No lesion (Score=0)	Lesions at inoc. Site (Score = 1-2)	Lesions at Distal Site (Score = 3-4)
0	7/7	0/7	0/7	0	7/7	0/7	0/7	0	3/3	0/3	0/3	0	3/3	0/3	0/3
1	2/7	5/7	0/7	1	7/7	0/7	0/7	1	3/3	0/3	0/3	1	3/3	0/3	0/3
2	0/7	7/7	0/7	2	7/7	0/7	0/7	2	3/3	0/3	0/3	2	3/3	0/3	0/3
3	0/5	5/5	0/5	3	5/5	0/5	0/5	3	2/3	1/3	0/3	3	3/3	0/3	0/3
4	0/5	5/5	0/5	4	3/5	2/5	0/5	4	2/3	1/3	0/3	4	3/3	0/3	0/3
5	0/5	3/5	2/5	5	2/5	3/5	0/5	5	1/3	0/3	2/3	5	3/3	0/3	0/3
6	0/5	4/5	1/5	6	3/5	2/5	0/5	6	1/3	0/3	2/3	6	3/3	0/3	0/3
7	0/5	3/5	2/5	7	3/5	2/5	0/5	7	0/3	0/3	3/3	7	3/3	0/3	0/3
8	0/5	4/5	1/5	8	3/5	2/5	0/5	8	1/3	0/3	2/3	8	3/3	0/3	0/3
9	1/5	3/5	1/5	9	2/5	0/5	3/5	9	1/3	0/3	2/3	9	3/3	0/3	0/3
10	2/5	2/5	1/5	10	1/5	1/5	3/5	10	1/3	0/3	2/3	10	3/3	0/3	0/3
11	3/3	0/3	0/3	11	1/3	0/3	2/3	11	1/3	1/3	1/3	11	3/3	0/3	0/3
12	3/3	0/3	0/3	12	1/3	0/3	2/3	12	1/3	0/3	2/3	12	3/3	0/3	0/3
13	3/3	0/3	0/3	13	1/3	0/3	2/3	13	1/3	1/3	1/3	13	3/3	0/3	0/3
14	3/3	0/3	0/3	14	1/3	0/3	2/3	14	1/3	0/3	2/3	14	2/3	0/3	1/3**
15	3/3	0/3	0/3	15	2/3	0/3	1/3	15	1/3	0/3	2/3	15	3/3	0/3	0/3
16	3/3	0/3	0/3	16	2/3	1/3	0/3	16	1/3	0/3	2/3	16	3/3	0/3	0/3
17	3/3	0/3	0/3	17	2/3	1/3	0/3	17	1/3	0/3	2/3	17	3/3	0/3	0/3
18	3/3	0/3	0/3	18	2/3	1/3	0/3	18	2/3	0/3	1/3	18	3/3	0/3	0/3
19	3/3	0/3	0/3	19	2/3	1/3	0/3	19	2/3	0/3	1/3	19	3/3	0/3	0/3
20	3/3	0/3	0/3	20	2/3	1/3	0/3	20	2/3	0/3	1/3	20	3/3	0/3	0/3
21	3/3	0/3	0/3	21	2/3	1/3	0/3	21	2/3	0/3	1/3	21	3/3	0/3	0/3

Table S2. Proportion of animals with lesions on each day including clinical lesion scores. Pigs were observed daily for the appearance of vesicular skin lesions. The proportion of pigs in each group with no lesions (score 0), lesions at the nasal planum inoculation site (lesion diameter < 2 cm – score 1; lesion diameter > 2cm – score 2) or at a distal site (lesion diameter < 2 cm – score 3; lesion diameter > 2cm – score 4). All distal site lesions were located on the feet. **One of the VSV-EBOV-contact control pigs (#10) showed a focal reddening (< 2 cm) of a hind limb on 14 days post inoculation (DPI), however it did not show ulcerative or vesicular features characteristic of VS and was ultimately determined to be a mechanical injury.

	Lesion Swabs			
Group	ID	DPI	Lesion Site	CN / g
wtVSV	8	3	Nasal Planum	$1.56x10^3$
wtVSV	17	3	Nasal Planum	$1.94x10^4$
wtVSV	18	2	Nasal Planum	$2.12x10^6$
wtVSV	18	3	Nasal Planum	$3.79x10^5$
wtVSV	18	5	Nasal Planum	$8.50x10^3$
wtVSV	20	3	Nasal Planum	$2.03x10^6$
wtVSV-contact	11	5	Nasal Planum	$1.44x10^6$
wtVSV-contact	11	6	Nasal Planum	$7.18x10^5$
wtVSV-contact	24	5	Left Hind Foot	$1.15x10^4$
VSV-EBOV	1	9	Left Hind Foot	$3.87x10^4$
VSV-EBOV	1	11	Left Hind Foot	$2.06x10^5$
VSV-EBOV	15	9	Left Front Foot	$2.98x10^5$
VSV-EBOV	15	10	Left Front Foot	$3.63x10^4$
VSV-EBOV	16	9	Right Hind Foot	$8.35x10^2$
VSV-EBOV	16	9	Left Hind Foot	$1.94x10^4$
VSV-EBOV-contact	19	10	Front Right Foot	ND

Table S3. RT-qPCR testing of selected lesion swabs for viral RNA. A swab from a lesion suspected to be secondary to incidental trauma in one VSV-EBOV-contact control pig (#19) confirmed no evidence of VSV-specific RNA. ID – pig identification; DPI – Days post inoculation; CN – copy numbers; ND – not detected.

		Nasal Swabs						Tonsilar Swabs					
**Group**	**ID**	**0 DPI**	**1 DPI**	**3 DPI**	**5 DPI**	**10 DPI**	**21 DPI**	**0 DPI**	**1 DPI**	**3 DPI**	**5 DPI**	**10 DPI**	**21 DPI**
wtVSV	3	0	0	0*	---	---	---	0	0	0*	---	---	---
wtVSV	6	0	0	< LOQ	$1.57x10^{3}$	< LOQ	---	0	$8.73x10^{2}$	< LOQ	$2.04x10^{7}$	0	---
wtVSV	8	0	0	$1.36x10^{8}$	$2.22x10^{5}$	0	---	0	0	$2.70x10^{4}$	$1.14x10^{3}$	0	---
wtVSV	9	0	$1.45x10^{6}$	0*	---	---	---	0	0	0*	---	---	---
wtVSV	17	0	0	$1.86x10^{3}$	$9.80x10^{4}$	0	0	0	0	$1.58x10^{3}$	$5.71x10^{5}$	0	0
wtVSV	18	0	0	$1.81x10^{6}$	$7.10x10^{5}$	0	0	0	0	$2.38x10^{3}$	$2.68x10^{5}$	0	< LOQ
wtVSV	20	0	0	$9.93x10^{4}$	$2.33x10^{6}$	0	0	0	0	$3.75x10^{5}$	$1.27x10^{4}$	0	0
wtVSV-contact	11	0	0	0	$4.86x10^{3}$	< LOQ	0	0	0	< LOQ	$2.72x10^{4}$	$2.22x10^{5}$	0
wtVSV-contact	12	0	0	< LOQ	< LOQ	0	0	0	0	$1.38x10^{4}$	$5.22x10^{6}$	0	< LOQ
wtVSV-contact	24	0	0	0	0	0	0	0	0	0	0	0	0
VSV-EBOV	1	0	0	0	< LOQ	0	0	0	0	0	0	< LOQ	0
VSV-EBOV	5	0	0	0*	---	---	---	0	0	0*	---	---	---
VSV-EBOV	7	0	0	0	$3.17x10^{4}$	< LOQ	---	0	0	0	0	< LOQ	---
VSV-EBOV	13	0	0	0	0	0	0	0	0	0	0	0	0
VSV-EBOV	15	0	0	0	$1.37x10^{6}$	0	---	0	0	0	0	0	---
VSV-EBOV	16	0	0	$8.60x10^{3}$	$9.80x10^{4}$	0	0	0	0	0	0	0	0
VSV-EBOV	21	0	< LOQ	$2.46x10^{6}$*	---	---	---	0	0	0*	---	---	---
VSV-EBOV-contact	4	0	0	0	< LOQ	0	0	0	0	0	0	0	0
VSV-EBOV-contact	10	0	0	0	0	< LOQ	0	0	0	0	0	0	0
VSV-EBOV-contact	19	0	0	< LOQ	0	0	0	0	0	0	0	0	0
Negative control	2	0	< LOQ	0*	---	---	---	0	0	0*	---	---	---
Negative control	14	0	< LOQ	0	< LOQ	0	0	0	0	0	< LOQ	0	0
Negative control	22	0	0	0	0	0	---	0	0	0	0	0	---
Negative control	23	0	0	0	0	0	0	0	0	0	0	0	0

Table S4. RT-qPCR testing of nasal and tonsillar swabs for viral RNA. All values are reported as copy number/mL. Crossed out boxes indicate the pig was euthanized prior to the collection day. ID – pig identification; DPI – days post inoculation; 0 – negative; < LOQ – Less than Limit of Quantitation (2 gene copies (gc) / μL of RNA for non-qualified tissues), treated as negative for analysis; * - sample was collected on 2 DPI prior to necropsy.

		Sera					
Group	ID	0 DPI	1 DPI	3 DPI	5 DPI	10 DPI	21 DPI
wtVSV	3	0	< LOQ	0*	---	---	---
wtVSV	6	0	0	0	0	0	---
wtVSV	8	0	0	0	0	0	---
wtVSV	9	0	< LOQ	0*	---	---	---
wtVSV	17	0	0	0	0	0	0
wtVSV	18	0	0	0	0	0	0
wtVSV	20	0	0	0	0	0	0
wtVSV-contact	11	0	0	< LOQ	0	0	0
wtVSV-contact	12	0	0	0	0	0	0
wtVSV-contact	24	0	0	< LOQ	0	0	0
VSV-EBOV	1	0	< LOQ	0	< LOQ	0	0
VSV-EBOV	5	0	0	0*	---	---	---
VSV-EBOV	7	0	0	0	0	0	---
VSV-EBOV	13	0	0	< LOQ	0	0	0
VSV-EBOV	15	0	< LOQ	0	0	0	---
VSV-EBOV	16	< LOQ	0	< LOQ	< LOQ	0	0
VSV-EBOV	21	0	0	0*	---	---	---
VSV-EBOV-contact	4	0	0	0	0	0	0
VSV-EBOV-contact	10	0	0	0	0	0	0
VSV-EBOV-contact	19	0	0	0	0	0	0
Negative control	2	0	0	0*	---	---	---
Negative control	14	0	0	0	0	0	0
Negative control	22	0	0	0	0	0	---
Negative control	23	0	0	0	0	0	0

Table S5. RT-qPCR testing of sera samples for viral RNA. Crossed out boxes indicate the pig was euthanized prior to that collection day. ID – pig identification; DPI – days post inoculation; 0 – negative; < LOQ – Less than Limit of Quantitation (2 gene copies / μL of RNA for non-qualified tissues), treated as negative for analysis; * - sample was collected on 2 DPI prior to necropsy.

Group	ID	DPI	Nasal Planum	Tonsil	Parotid-LN	Retropharyngeal -LN	Superficial Cervical-LN	Mandibular -LN	Mammary Gland	Gluteal Muscle	Peyer's Patch	Spleen	Testicle	Urinary Bladder	Parotid Salivary Gland
wtVSV	3	2	$1.47x10^7$	<LOQ	$6.45x10^4$	$7.30x10^3$	$4.14x10^4$	$8.17x10^4$	NC	NC	NC	NC	NC	NC	NC
wtVSV	9	2	$3.23x10^9$	$2.30x10^6$	$5.45x10^7$	$5.22x10^4$	$1.57x10^5$	$1.42x10^4$	NC	NC	NC	NC	NC	NC	NC
wtVSV	6	10	$1.07x10^5$	$1.98x10^7$	$8.21x10^5$	0	< LOQ	$5.23x10^4$	NC	NC	NC	NC	NC	NC	NC
wtVSV	8	10	$5.50x10^4$	< LOQ	< LOQ	< LOQ	$1.05x10^6$	$3.32x10^5$	NC	NC	NC	NC	NC	NC	NC
wtVSV	17	21	0	$4.94x10^6$	$4.91x10^7$	$2.59x10^4$	$3.42x10^4$	$3.95x10^6$	NC	NC	NC	NC	NC	NC	NC
wtVSV	18	21	0	$1.59x10^9$	$9.07x10^5$	$2.67x10^4$	0	$1.37x10^5$	NC	NC	NC	NC	NC	NC	NC
wtVSV	20	21	< LOQ	<LOQ	$4.44x10^6$	$2.73x10^4$	0	$1.20x10^8$	NC	NC	NC	NC	NC	NC	NC
wtVSV-contact	11	21	0	$2.99x10^8$	0	$2.39x10^4$	$5.19x10^6$	$4.53x10^7$	0	0	0	0	0	0	0
wtVSV-contact	12	21	0	< LOQ	0	< LOQ	0	0	$4.53x10^7$	0	0	0	0	0	0
wtVSV-contact	24	21	0	0	$1.19x10^4$	0	0	< LOQ	0	0	0	$4.53x10^7$	NC	0	0
VSV-EBOV	5	2	$1.50x10^9$	0	0	$2.34x10^5$	$6.36x10^5$	0	0	$1.45x10^5$	$2.43x10^5$	0	$1.10x10^5$	0	0
VSV-EBOV	21	2	$2.61x10^8$	< LOQ	$5.04x10^7$	$1.65x10^5$	$4.52x10^5$	$1.21x10^6$	0	0	0	< LOQ	NC	0	0
VSV-EBOV	7	10	<LOQ	0	$6.10x10^4$	$2.20x10^8$	$4.73x10^5$	$8.93x10^6$	0	< LOQ	0	0	NC	0	$3.45x10^7$
VSV-EBOV	15	10	$1.86x10^4$	< LOQ	$8.81x10^8$	$2.59x10^7$	$1.40x10^6$	$1.26x10^6$	0	0	0	<LOQ	$2.83x10^4$	$2.34x10^4$	$5.87x10^4$
VSV-EBOV	1	21	< LOQ	0	$2.95x10^6$	$1.17x10^6$	< LOQ	$3.87x10^3$	0	0	0	0	0	0	0
VSV-EBOV	13	21	0	0	0	$2.20x10^4$	$5.92x10^3$	0	0	0	0	0	0	0	0
VSV-EBOV	16	21	< LOQ	0	$8.90x10^7$	$8.15x10^5$	0	$1.68x10^4$	0	0	0	$9.17x10^3$	NC	0	0
VSV-EBOV-contact	4	21	0	0	0	0	< LOQ	0	0	0	0	0	NC	0	0
VSV-EBOV-contact	10	21	< LOQ	0	0	0	0	0	0	0	0	0	0	0	0
VSV-EBOV-contact	19	21	0	0	0	0	0	0	0	0	0	0	NC	0	0
Negative control	2	2	$9.47x10^3$	0	0	0	0	0	0	0	0	0	0	0	0
Negative control	14	10	0	0	0	0	0	0	0	0	0	0	0	0	0
Negative control	22	21	0	0	0	0	0	0	0	0	0	0	NC	0	0
Negative control	23	21	0	0	0	0	0	0	0	0	0	0	0	0	0

Table S6.RT- qPCR testing of selected tissue samples collected at necropsy for viral RNA. Nasal planum samples include any lesions that were present at the time of necropsy. All values are reported as copy number/g. Crossed out boxes indicate the pig has been euthanized prior to that collection day. LN – lymph node; NC – samples not collected; ID – pig identification; DPI – days post inoculation; 0 – negative; < LOQ – Less than Limit of Quantitation (2gc / µL of RNA for non-qualified tissues), treated as negative for analysis. The result from the nasal planum tissue of control Pig #2 was the only positive result from any negative control sample throughout the entire study and is therefore considered a false positive, likely due to contamination.

	Lesion Tissue Samples			
**Group**	**ID**	**DPI**	**Lesion Site**	**CN / g**
wtVSV	6	10	Left Front Foot	1.61×10^{5}
wtVSV	8	10	Left Front Phalangeal Joint	5.79×10^{5}
wtVSV	8	10	Left Front Lateral Accessory Digit	2.27×10^{11}
wtVSV	8	10	Left Front Foot	1.66×10^{4}
wtVSV	8	10	Left Front Foot	1.24×10^{4}
wtVSV-contact	24	21	Right Hind Foot	2.88×10^{5}
wtVSV-contact	24	21	Right Hind Foot	4.88×10^{5}
wtVSV-contact	24	21	Right Hind Foot	5.30×10^{9}
VSV-EBOV	1	21	Left Hind Foot	4.92×10^{4}

Table S7. RT-qPCR testing of selected lesion tissues for viral RNA. ID – pig identification; DPI – days post inoculation; CN – copy numbers.

Group	ID	0 DPI	21 DPI
wtVSV	17	< 20	10,240
wtVSV	18	< 20	2,256
wtVSV	20	< 20	2,256
wtVSV-contact	11	< 20	20,480
wtVSV-contact	12	< 20	10,240
wtVSV-contact	24	< 20	10,240
VSV-EBOV	1	< 20	< 20
VSV-EBOV	13	< 20	< 20
VSV-EBOV	16	< 20	< 20
VSV-EBOV-contact	4	< 20	< 20
VSV-EBOV-contact	10	< 20	< 20
VSV-EBOV-contact	19	< 20	< 20
Negative control	14	< 20	< 20
Negative control	23	< 20	< 20

Table S8. VSV serum neutralization titers of study pigs, determined pre-challenge (0 DPI) and at the conclusion of the study (21 DPI).

Group	ID	0 DPI	10* or 21 DPI
VSV-EBOV	1	< 1:20	405
VSV-EBOV	7	< 1:20	84*
VSV-EBOV	13	< 1:20	565
VSV-EBOV	15	< 1:20	325*
VSV-EBOV	16	< 1:20	494
VSV-EBOV-contact	4	< 1:20	< 1:20
VSV-EBOV-contact	10	< 1:20	< 1:20
VSV-EBOV-contact	19	< 1:20	< 1:20
Negative control	14	< 1:20	< 1:20
Negative control	23	< 1:20	< 1:20

Table S9. $PRNT_{60}$ titers of study pigs against VSV-EBOV pre-challenge (0 DPI) and at day 10 (10 DPI) or the conclusion of the study (21 DPI).

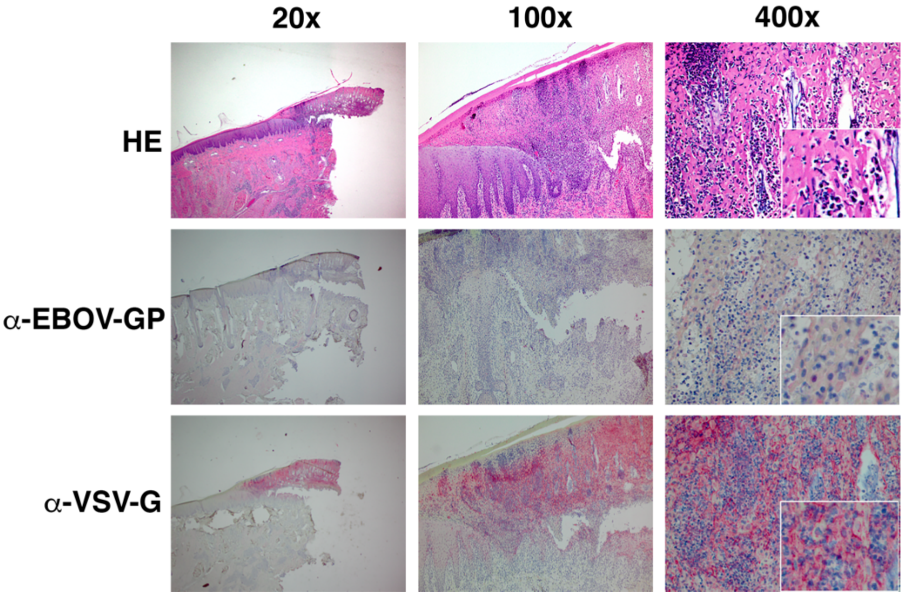

Figure S1. Immunohistochemical (IHC) analysis of wtVSV-infected Pig #3 nasal planum lesion. Lesion tissue from the nasal planum of wtVSV-infected Pig #3 was collected on scheduled necropsy 2 DPI. Hematoxylin and Eosin (HE) staining shows a focal ruptured vesicle in the stratum spinosum expanding and splitting the epidermis. IHC analysis with anti-EBOV-GP rabbit polyclonal antibody shows weak non-specific reactivity indicating no positive immunostaining. IHC with anti-VSV-G rabbit polyclonal antibody shows positive (red) immunostaining in the stratum spinosum and granulosum of the epidermis, indicating the presence of VSV G protein.

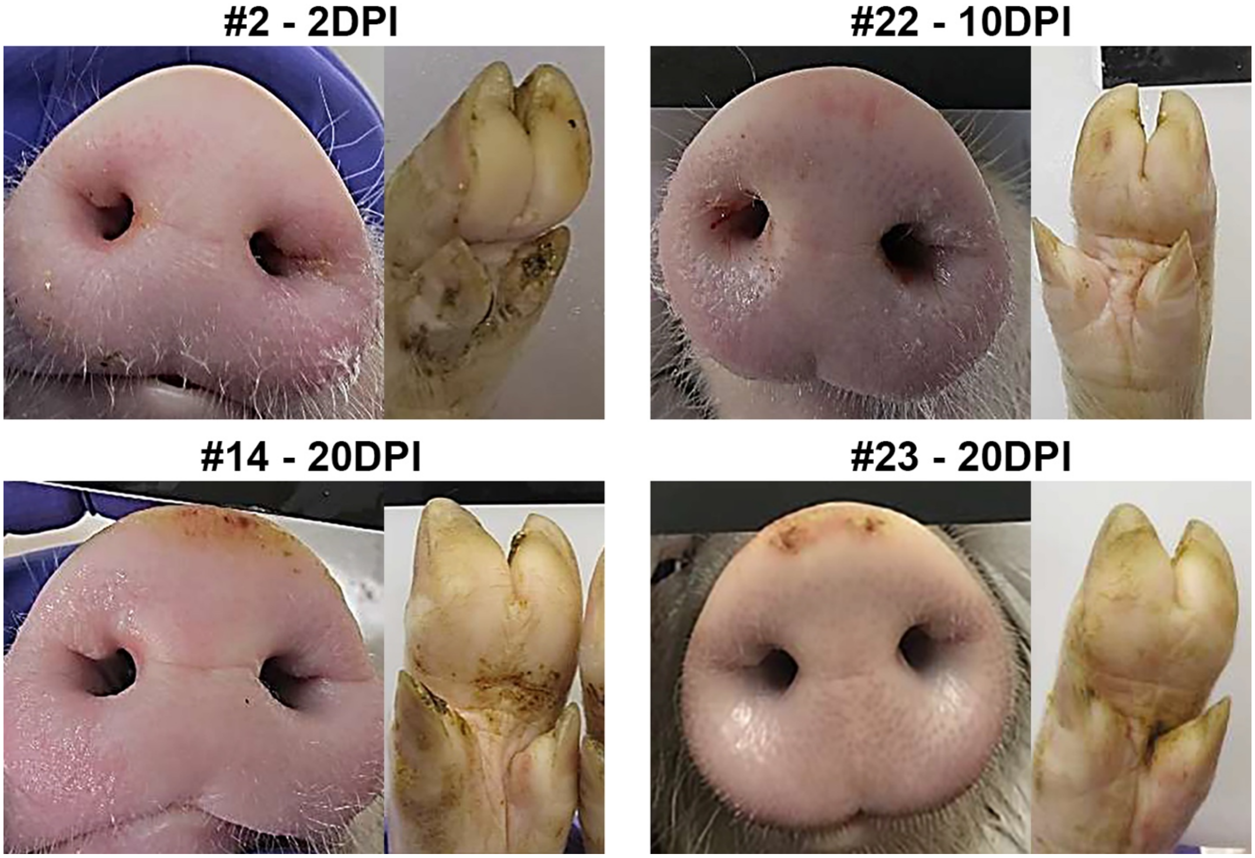

Figure S2. Representative pictures showing a lack of vesicular lesions in uninoculated negative control pigs. Snout and right front ventral foot are shown.

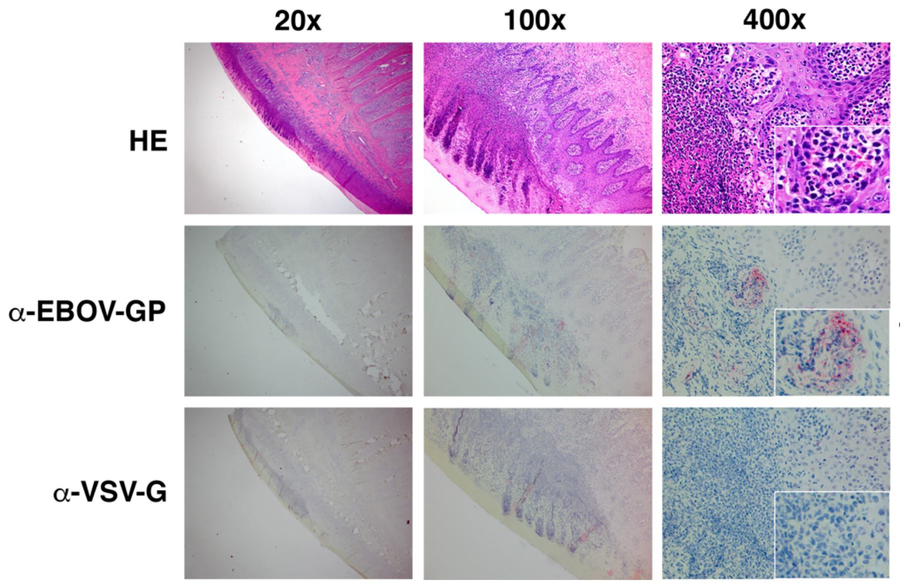

Figure S3. Immunohistochemical (IHC) analysis of VSV-EBOV-infected Pig #5 nasal planum lesion. Lesion tissue from the nasal planum of VSV-EBOV-infected Pig #5 was collected on scheduled necropsy 2 DPI. Hematoxylin and Eosin (HE) staining shows a focally extensive area of coagulative necrosis and inflammation in the stratum spinosum of the epidermis. IHC analysis with anti-EBOV-GP rabbit polyclonal antibody shows multifocal positive (red) staining in the stratum spinosum of the epidermis indicating the presence of EBOV GP protein. IHC with anti-VSV-G rabbit polyclonal shows weak, non-specific staining indicating an absence of the VSV-G protein.

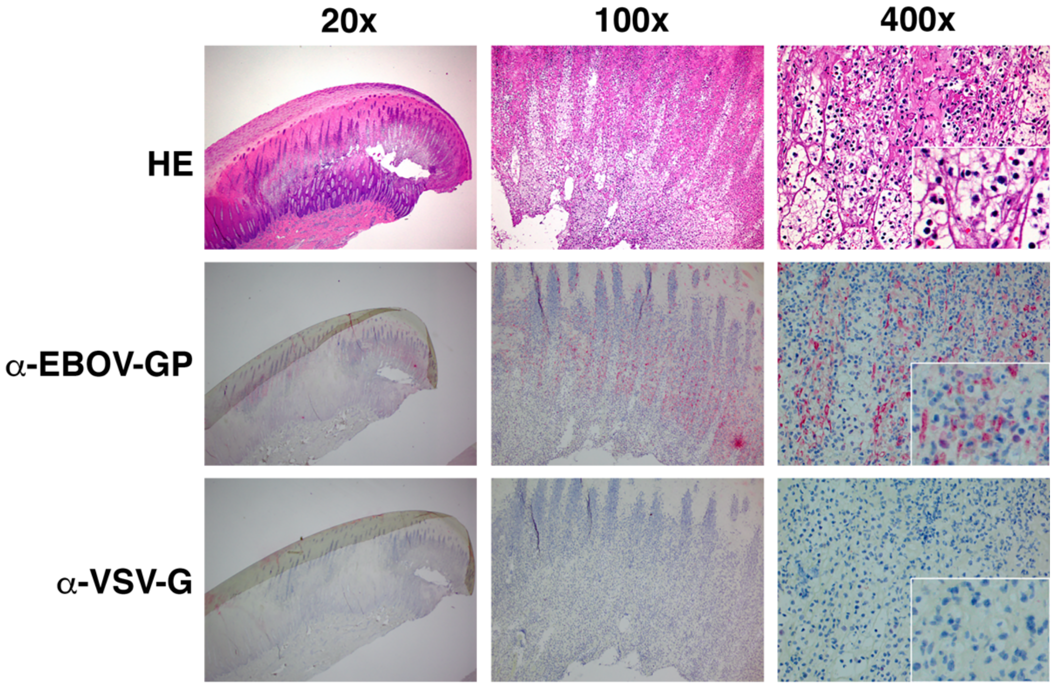

Figure S4. Immunohistochemical (IHC) analysis of VSV-EBOV-infected Pig #15 foot (hoof and skin). Lesion tissue from the foot (hoof and skin) of VSV-EBOV-infected Pig #15 was collected on scheduled necropsy 10 DPI. Hematoxylin and Eosin (HE) staining shows a focal vesicle in the stratum spinosum expanding and splitting the epidermis. IHC analysis with anti-EBOV-GP rabbit polyclonal antibody shows widespread strong positive (red) granular staining in the stratum spinosum and granulosum of the epidermis indicating the presence of Ebola virus GP protein. IHC with anti-VSV-G rabbit polyclonal shows weak, non-specific staining indicating an absence of the VSV-G protein.
